# Bcl-3 regulates the function of Th17 cells through raptor mediated glycolysis metabolism

**DOI:** 10.3389/fimmu.2022.929785

**Published:** 2022-09-09

**Authors:** Hui Liu, Lin Zeng, Yang Yang, Zhen Huang, Chunlei Guo, Liwenhui Huang, Xinqing Niu, Chenguang Zhang, Hui Wang

**Affiliations:** ^1^ Henan Key Laboratory of Immunology and Targeted Drug, Henan Collaborative Innovation Center of Molecular Diagnosis and Laboratory Medicine, School of Laboratory Medicine, Xinxiang Medical University, Xinxiang, China; ^2^ Department of Translational Medicine Center, The First Affiliated Hospital of Zhengzhou University, Zhengzhou, China

**Keywords:** Bcl-3, Th17, lactate, Raptor, glycolysis metabolism

## Abstract

Bcl-3 is an atypical IκB family member that regulates transcription in the nucleus by binding to the p50/p52 homologous dimer subunit. Although various studies illustrate the important role of Bcl-3 in physiological function, its role in metabolism is still unclear. We found that Bcl-3 has a metabolic regulatory effect on autoimmunity. Bcl-3-depleted mice are unable to develop experimental autoimmune encephalomyelitis. The disease resistance was linked to an increase in lactate levels in Th17 cells, and lactate could alleviate EAE development in WT mice. Bcl-3 deficient mice had more differentiated Th17 cells and an increased extracellular acidification rate in these cells. Concurrently, their ultimate respiration rate and respiratory reserve capacity were significantly lower than wild-type mice. However, adding GNE-140 (LADH inhibitor) to Bcl-3-deficient Th17 cells could reverse the phenomenon, and lactate supplementation could increase the glycolysis metabolism of Th17 cells in WT mice. Mechanically, Bcl-3 could interact with Raptor through ANK and RNC domains. Therefore, Bcl-3 regulates Th17 pathogenicity by promoting Raptor mediated energy metabolism, revealing a novel regulation of adaptive immunity.

## Introduction

The nuclear factor kappa-B (NF-κB) transcription factor family regulates the expression of numerous genes and critical biological processes (1). The IκB family proteins regulated the activity of NF-κB factors (2). Bcl-3 is an atypical member of IκB protein family that regulates NF-κB activity and facilitates or suppresses the expression of NF-κB target genes. Depending upon the stimuli and cell type, it regulates various cellular functions and processes, including cell proliferation and differentiation, apoptosis induction, and various immune responses (3–5). Furthermore, Bcl-3 is a regulator of context-dependent cellular responses in B cell development and Th cell differentiation, survival, and proliferation (6–8). Bcl-3 regulates Th cell plasticity by preventing c-Rel and p65 binding to NF-κB binding sites, thereby maintaining the Th1 cell phenotype and preventing it from converting to Th17-like cells (9). Bcl-3 can accelerate Th2 cell differentiation by activating GATA3 transcription (8). However, the conditional overexpression of Bcl-3 in mice T cells results in impaired development of Th1, Th2, and Th17 cells and dysregulation of Treg function (10). However, the specific modulatory role of Bcl-3 on the differentiation of Th cells remains elusive.

Bcl-3 plays an important role in inflammation. On the one hand, it can act as an anti-inflammatory factor, preventing the onset and progression of inflammation. For example, it controls the inflammatory response against invaded microbes by regulating the synthesis of cytokines, such as IL-10, IFN-γ, and TNF-α (11, 12). It can prevent acute inflammatory lung injury in mice by restraining granulocyte production in inflammatory conditions (13). Bcl-3 can also prevent the development of TNF-α dependent lupus phenotype of BL6/LPR mice (14). On the other hand, it acts as a pro-inflammatory factor during the inflammation process. For example, it indirectly contributes to the progression of rheumatoid arthritis by producing IL-21 (15). In nonalcoholic steatohepatitis, hepatocyte-specific Bcl-3 can impair hepatic glucose and lipid metabolism, promoting hepatic steatosis and inflammation (16). Mice with T-cell-specific overexpression of Bcl-3 induced defective development and function of Tregs, aggravating colitis (17). Bcl-3-depleted T cells cannot induce transfer-induced colitis and experimental autoimmune encephalomyelitis (EAE), caused by the decrease of GM-CSF in Th1 and an increase in Th17 cells (9). However, Bcl-3 overexpression in mice had impaired Th17 cells development leading to EAE resistance (10). Therefore, the role of Bcl-3 is not unilateral in the inflammatory process.

Recent studies indicate a dynamic and precise relationship between metabolic processes and specific cellular functions maintained during the immune response process. T cell activation and differentiation are associated with metabolic reorganization. In comparison, aerobic glycolysis is considered a metabolic hallmark of T cells activation (18). However, the effect of this aerobic glycolysis on T cell responses is unclear. Our previous studies indicate that Bcl-3 is a new metabolic regulator that regulates lipid metabolism during obesity progression (19). Some other studies revealed depletion of Bcl-3 in hematopoietic cells could increase the risk of diabetes (20). These findings intricate the role of Bcl-3 in energy metabolism. But the exact mechanism of Bcl-3 in mediating energy metabolism remains unclear.

The present study shows that Bcl-3 regulates glycolysis metabolism in Th17 cells. We proved that Bcl-3 deficiency in Th17 cells increased glycolysis but blocked mitochondrial energy metabolism. It is evident from the results that Bcl-3 regulates lactate synthesis by interacting with Raptor, thus regulating the differentiation of Th17 cells that alleviated experimental autoimmune encephalomyelitis. The study may provide new insights into the inflammatory process by studying the effect of Bcl-3 on energy metabolism.

## Materials and methods

### Reagents

Recombinant mouse IL-6, purified anti-mouse CD28 mAb, anti-mouse CD3 mAb, FITC conjugated anti-mouse CD4, APC-conjugated anti-mouse IFN-γ, and PE-conjugated anti-mouse IL-17, anti-mouse IL-4 mAb, anti-mouse IFN-γ mAb and IL-17 ELISA kits were bought from BioLegend (San Diego, CA, USA), Recombinant human TGFβ1.2 was obtained from eBioscience (San Diego, CA, USA). L-lactate detection kit was obtained from Eton bioscience (San Diego, CA, USA). The LADH inhibitor GNE-140 was purchased from MCE (Minneapolis, MN). Pertussis toxin was obtained from List Biological Lab (Campbell, CA, USA). The glycolysis stress test kit and mitochondria stress test kit were obtained from Agilent Technologies (Palo Alto, Calif.). The myelin oligodendrocyte glycoprotein (MOG) 35-55 was purchased from GL Biochem Corporation (Shanghai, China). Iscove’s Modified Dulbecco Medium (IMDM), Dulbecco’s modified Eagle’s medium (DMEM) and fetal bovine serum (FBS) were ordered from Gibco (South Logan, UT, USA). Non-viable, desiccated Mycobacterium tuberculosis H37 RA and incomplete Freund adjuvant (IFA) was bought from BD Difco (Detroit, MI, USA); L-sodium lactate, Phorbol 12-myristate 13-acetate and Ionomycin were bought from Sigma Aldrich Company (St. Louis, MO).

### Animals

The Bcl3^-/-^ mice derived from C57/B6N was gifted by Prof. Yinming Liang (Xinxiang Medical University) using the CRISPR/Cas9 system. All mice were kept in a specific pathogen-free condition at the Animal Resource Center at Xinxiang Medical University. All experiments were conducted on female mice aged 6-8 weeks old and per Xinxiang Medical University’s animal care guidelines.

### Th17 cell culture

Naïve CD4^+^ T cells were isolated from the spleens of Bcl-3-/- or wild-type (WT) mice and sorted using naïve CD4^+^ T cells isolation kits (Biolegend). Flow cytometry was used to ensure the purity of cells. 10 µg/mL anti-CD3 in IMDM cell medium was pre-coated in a 48-well plate to differentiate Th17 cells. T cells were cultured with IMDM cell medium, supplemented with 10% FBS, 55 µM β mercaptoethanol, 100 U/mL penicillin, 100 mg/mL streptomycin, 1 µg/mL anti-CD28 and 2 ng/mL TGF-β, 10 ng/mL IL-6, 5 µg/mL anti-IL-4 and 5 µg/mL anti-IFN-γ. The cells were harvested after 3-4 days of incubation, followed by the supernatant collection to determine the secretion of IL-17 and lactate.

### Induction and assessment of EAE

EAE induction and evaluation were carried out according to the published guidelines (21). Briefly, 200 μg MOG35-55 peptide was emulsified in CFA containing 5 mg/mL non-viable and desiccated M. tuberculosis H37 RA before subcutaneously injected into Bcl-3-/- and WT mice. On days 0 and 2, 300 ng Pertussis toxin dissolved in PBS was injected intraperitoneally. Clinical evaluation of EAE scores was performed every other day as directed by the study protocol.

### Flow cytometry

Th17 cells or lymphocytes were isolated from the specific organs (spleen, lymph nodes, spinal cords) of the mice for intracellular cytokines staining. These isolated cells were stimulated for 4-6 h by Phorbol 12-myristate 13-acetate, Ionomycin, and Golgi plug for 4-6 h. Then, the cells were stained with surface antibodies, fixed, and permeabilized according to the study protocol. Finally, stained with intracellular cytokine antibodies and analyzed through a flow cytometer (BD Calibur).

### Histology

The lumbosacral portion of spinal cords was obtained on day 18 after immunization. These 5 mm thickness slices were embedded in paraffin after being fixed by 4% (w/v) paraformaldehyde. Finally, these slices were stained with hematoxylin and eosin (H&E) or Luxol fast blue (LFB) to analyze the inflammation or demyelination.

### Immunoblotting

The cells were lysed with a cell lysis buffer (Beyotime) having protease inhibitors cocktail tablets and phosphatase inhibitor cocktail (Roche), followed by determination of protein concentration using a BCA kit (Beyotime). The proteins were isolated by 10% SDS-PAGE gels and transferred to polyvinylidene difluoride membranes (Millipore). After incubating the membranes with antibodies, they were visualized using enhanced chemiluminescent HRP substrate (Millipore) and detected by Amersham Imager 600RGB detection system (GE Healthcare), as described previously (22).

### Transfection and co-IP assays

HEK293T cell line was cultured in complete DMEM supplemented with 10% FBS, penicillin (100 U/mL), and streptomycin (100 μg/mL). According to the manufacturer’s specifications, plasmids were transfected into HEK293T cells through liposome 2000. Forty-eight hours after transfection, the cells were collected and lysed by IP lysis buffer (beyotime) having protease inhibitor cocktail tablets (Roche) for the co-IP of Flag-Bcl-3 and HA-Raptor proteins. The lysates were cleared and mixed with 1 μg anti-Flag (Proteintech) or 1 μg anti-HA (A2095, Sigma) agarose beads (20 µL) (Santa Cruz) and incubated at 4°C overnight. Following the incubation, the beads were washed four times with lysis buffer and analyzed by Western Blot.

The cell lysates were incubated with anti-Bcl-3 antibody (16611, CST, 5 µg) or anti-Raptor antibody (24C12, CST, 5 µg) by the protocol mentioned above for the co-IP of endogenous Bcl-3 and Raptor. The mouse purified IgG was used as a negative control in the analysis.

### Metabolic assays

The cultured medium was collected after 72 h incubation to determine the lactate production of Th17 cells. The blank media without Th17 cells incubated in the same plate was considered to control. The lactate levels in culture media were determined with lactate assay kit II (Sigma).

The Oxygen Consumption Rate (OCR) and extracellular acidification rate (ECAR) were measured using an XF24 extracellular flux analyzer (Seahorse Bioscience) in accordance with the manufacturer’s protocol. Briefly, Th17 cells were seeded in XF24 microplates (3×10^5^cells/well) pre-treated by Cell-Tak adhesive (Corning) and cultured in a Seahorse XF DMEM supplemented with 10 mM glucose, 1.0 mM sodium pyruvate, and 2.0 mM L-glutamine for ECAR detection or 2.0 mM L-glutamine for OCR detection. The plates were then placed in a non-CO2 incubator for 25 min and fixed by quick centrifugation. After fixation, the cells were again incubated in a non-CO2 incubator for 30 min. Glycolysis was detected using the XF glycolysis stress test kit (Agilent). Following the three baseline readings, glucose (10 mM) and oligomycin (1 µM), which inhibited the production of mitochondrial ATP and switched energy production to glycolysis, were sequentially injected into the ports. The increased ECAR represents the maximum glycolytic capacity of T cells. Finally, the injection of 100 mM 2-DG, a glycolysis inhibitory glucose analog, and results in decreased ECAR, confirming that the observed ECAR was due to the glycolysis process. The OCR was detected under different experimental conditions using Mito stress test kit (Agilent), followed by oligomycin (1 µM), FCCP (1 µM) OCR, and rotenone/antimycin A (0.5 µM) injection. The data were analyzed using wave 2.4 software.

### Metabolomic analysis

The naïve CD4^+^ T cells were isolated from the spleen lymphocytes of WT and Bcl-3-/- mice. After centrifugation, cells were kept in liquid nitrogen for 30 s and stored under -80°C condition. Then, metabolites were immediately extracted and subjected to targeted metabolomic analysis for central carbon metabolism. Electrospray ionization mass spectrometry (cation) or Agilent 6460 Triple Quad LC/MS (anion) was conducted by Beijing Novogene Technology Co., LTD.

### RNA-seq

Total RNA was extracted using the TRIzol Reagen (invitrogen, 15596018) following the manufacturer’s protocol. RNA integrity was evaluated using the Agilent 2100 Bioanalyzer (Agilent Technologies, Santa Clara, CA, USA). The samples with RNA Integrity Number (RIN) ≥ 7 were subjected to the subsequent analysis. The libraries were constructed using TruSeq Stranded mRNA LTSample Prep Kit (Illumina, San Diego, CA, USA) according to the manufacturer’s instructions. The transcriptome sequencing and analysis were conducted by OE Biotech Co., Ltd. (Shanghai, China).

The libraries were sequenced by the Illumina sequencing platform (Illumina novaseq6000) and 150bp paired-end reads were generated. About 6.64-7.52 G raw reads for each sample was generated. Raw data (raw reads) of fastq format were firstly processed using Trimmomatic and the low-quality reads were removed to obtain the clean reads. Then about 38.85 G clean reads for each sample were retained for subsequent analyses. The clean reads were mapped to the mouse genome (GRCm38.p6) using HISAT2. FPKM of each gene was calculated using Cufflinks, and the read counts of each gene were obtained by HTSeq-count. Differential expression analysis was performed using the DESeq (1.18.0) R package. p value < 0.05 and fold change < 0.5 was set as the threshold for significantly differential expression. The accession number for the deposited RNA-seq data is: PRJNA855272.

### Quantitative RT-PCR

Total RNA was extracted by TRIzol Reagent (Invitrogen, Carlsbad, CA) and reverse-transcribed by Quantscript RT Kit (TAKARA). Real-time quantitative PCR was carried out with TB Green Premix Ex Taq II (Takara) and detected by an Applied Biosystems 7500 System. Concurrently, HPRT gene was considered an internal control gene. The relative expression was analyzed by the2−ΔΔct method. The primer sequences used were as follows:

For HPRT, 5’-TCAGTCAACGGGGGACATAAA-3’ (Forward), 5’-GGGGCTGTACTGCTTAACCAG-3’ (reverse), for LDHA, 5’-CAAAGACTACTGTGTAACTGCGA-3’ (forward) 5’-TGGACTGTACTTGACAATGTTGG-3’ (reverse), for MCT1, 5’-AGGTGCTCTTCATGTGCATTG-3’ (forward) and 5’-TGGAGGTAGACCTTCTTCACAC-3’ (reverse)

### Statistical analysis

All results were presented as mean ± SD, except the score of EAE model, which was presented as mean ± SEM. Two-tailed Student’s t-test analyzed the differences among the study groups, and Kruskal-Wallis test analyzed EAE score curves. All statistical data were analyzed using Prism 8.0 for Windows (GraphPad Software). P<0.05 was considered statistically significant.

## Results

### Loss of Bcl-3 increased percentage of Th17 cells

**Figure 1:**
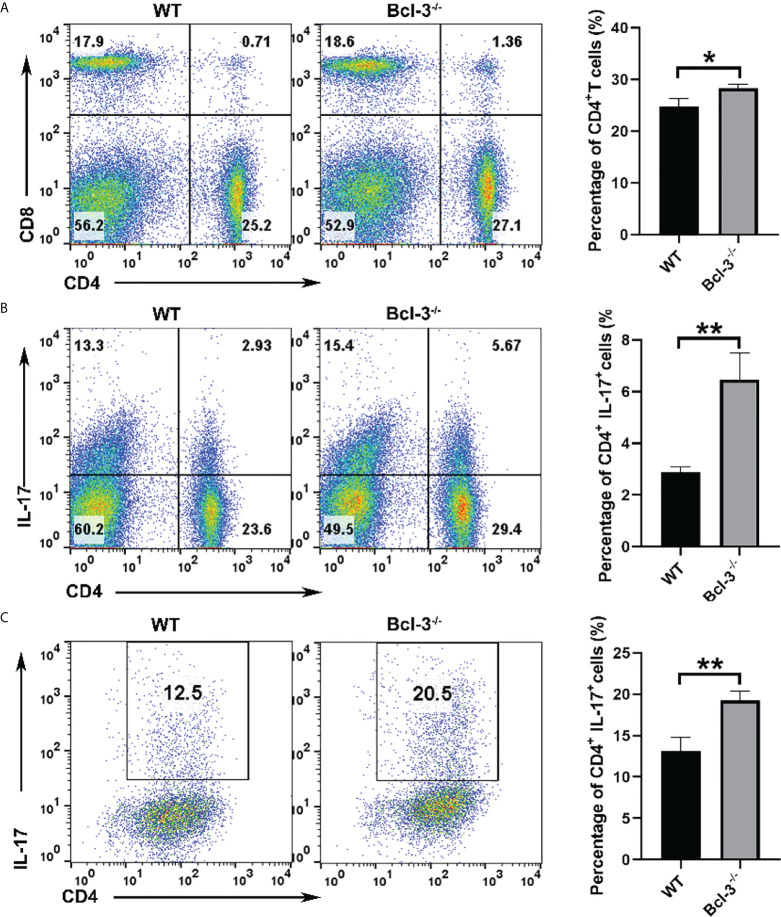
Loss of Bcl-3 diminished numbers of CD4+ T cells and increased numbers of Th17 cells. Lymphocytes were isolated from spleen of Bcl-3^-/-^ and WT mice and analyzed by FACS. **(A)** Flow cytometry plots gated on CD4^+^ T cells and statistical analysis. **(B)** Percentage and statistical analysis of IL-17^+^CD4^+^ T cells. **(C)** Differentiation Th17 cells were induced using *in vitro* polarization assay, and the percentage of Th17 cells were detected by FACS. n = 3 for each group. Data above are representative of 3 independent experiments. *P <0.05, **P <0.01.

Bcl-3 was known as a bidirectional regulator of inflammation, with the ability to inhibit Th17 plasticity while promoting pathogenicity by limiting the transformation of Th1 cells into Th17-like cells. To assess the roles of Bcl-3 on mice T cells, we isolated CD4^+^ T cells and Th17 cells from the spleen of WT and Bcl-3^-/-^ mice. It was found that there was no significant difference in the percentage of CD8^+^ T cells in the spleen, but the percentage of CD4^+^ T cells in the spleen of Bcl-3^-/-^ mice was slightly increased (**Figure 1A**). Furthermore, we investigated the IL-17 production of CD4^+^ T cells. The results indicate that Bcl-3 depletion increased the percentage of IL-17^+^ CD4^+^ T cells relative to WT mice, as shown in **Figure 1B**. However, we found that the thymus derived IL-17^+^ CD4^+^ T cells have no difference in Bcl-3^-/-^ mice and WT mice. These findings revealed that Bcl-3 depletion could upregulate the secretion of IL-17 in peripheral CD4^+^ T cells (**Supplementary Figure S1A**). The effect of Bcl-3 depletion on IL-17 production was verified by detecting the expression of Th17 transcription factor RORγt in the blood and spleen of WT and Bcl-3^-/-^ mice. The results indicate that Bcl-3 depletion causes a significant increase in RORγt expression in blood and spleen (**Supplementary Figure S1B**). The above findings suggest that Bcl-3 can influence IL-17 expression, which might affect Th17 differentiation. We investigated the role of Bcl-3 in the development of Th17 cells using an *in vitro* polarization assay to demonstrate the role of Bcl-3 in Th17 differentiation process. The CD4^+^CD44^low^ CD62L^high^ cells were considered as naive CD4^+^ T cells and the post-sort purity of naive CD4^+^ T cells is about 80%-90% (**Supplementary Figure S2**). The results illustrate that naive CD4^+^ T cells derived from Bcl-3 depleted mice differentiate into IL-17 producing cells more than those derived from WT mice (**Figure 1C**). These findings show that Bcl-3 depletion promotes Th17 cell development in both naive mice and culture.

### Bcl-3 depletion promotes the glycolysis and inhibits the mitochondrial respiration

**Figure 2:**
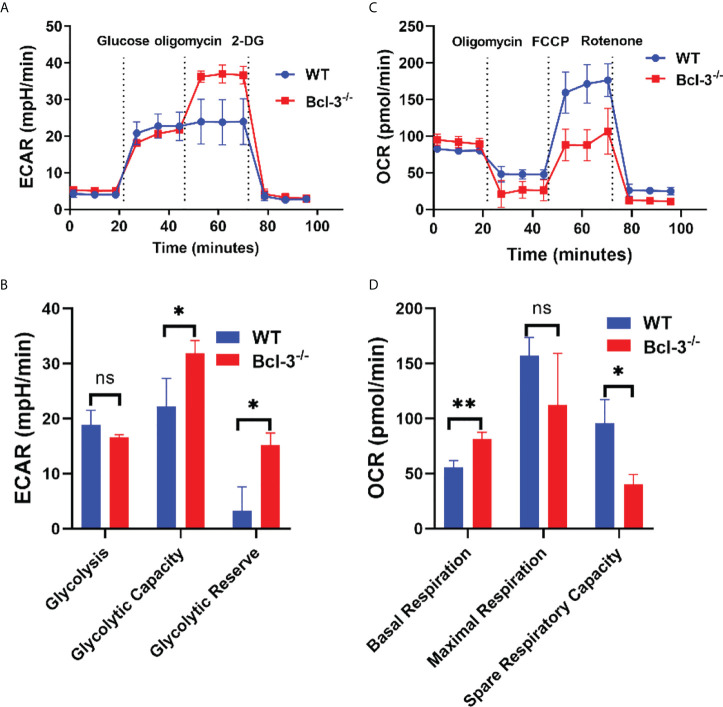
Bcl-3 depletion promotes glycolysis and inhibits mitochondrial respiration of Th17 cells. Naïve CD4^+^ T cells were isolated from the spleen of Bcl-3^-/-^ and WT mice and induced Th17 differentiation by *in vitro* polarization assay and detected the metabolic signals by seahorse XF24 extracellular flux analyzer. **(A)** Glycolytic pathway was detected by extracellular acidification rate (ECAR). **(B)** Statistical analysis of ECAR **(C)** mitochondrial respiration was detected by oxygen consumption rate (OCR). **(D)** Statistical analysis of OCR. N = 3 for each group. Data above are representative of 3 independent experiments. *P <0.05, **P <0.01.

It was previously established that glycolysis plays a role in T cell activation and differentiation. A glycolysis metabolite, the lactate plays an important role in T cells development, proliferation and function. To further investigate the mechanism by which Bcl-3 regulates Th17 cells differentiation, we explored the influence of Bcl-3 on the energy metabolism of Th17 cells. We used an *in vitro* polarization assay to induce Th17 differentiation and a seahorse XF24 extracellular flux analyzer to detect metabolic signals. Oxygen consumption rate (OCR) was used to detect mitochondrial respiration, and extracellular acidification rate (ECAR) was utilized to detect the glycolytic pathway. The results revealed that extracellular acidification was faster in the Bcl-3^-/-^ group than in WT group (**Figures 2A, B**). However, there was no significant difference in the basic aerobic respiration rate in Bcl-3 knockout Th17 cells and WT mice. However, the ultimate respiration rate and respiratory reserve capacity induced by Carbonyl cyanide 4-(trifluoromethoxy) phenylhydrazone Ready Made Solution (FCCP) were significantly lower (**Figures 2C, D**). These findings implicit the role of Bcl-3 in the mitochondrial energy metabolism of Th17 cells.

### Bcl-3 regulates the differentiation of Th17 cells by lactate

We isolated and purified the naïve CD4^+^ T cells from the spleen lymphocytes of WT and Bcl-3^-/-^ mice for targeted glycometabolomic detection and analysis to investigate the underlying mechanism of Bcl-3 on energy metabolism and Th17 cell differentiation. The data indicate that the lactic acid (LA) contents were significantly varied in 30 components associated with glucose metabolism. It was found that these contents were considerably higher in Bcl-3^-/-^ mice than in normal WT mice (**Supplementary Figure S3**).

**Figure 3:**
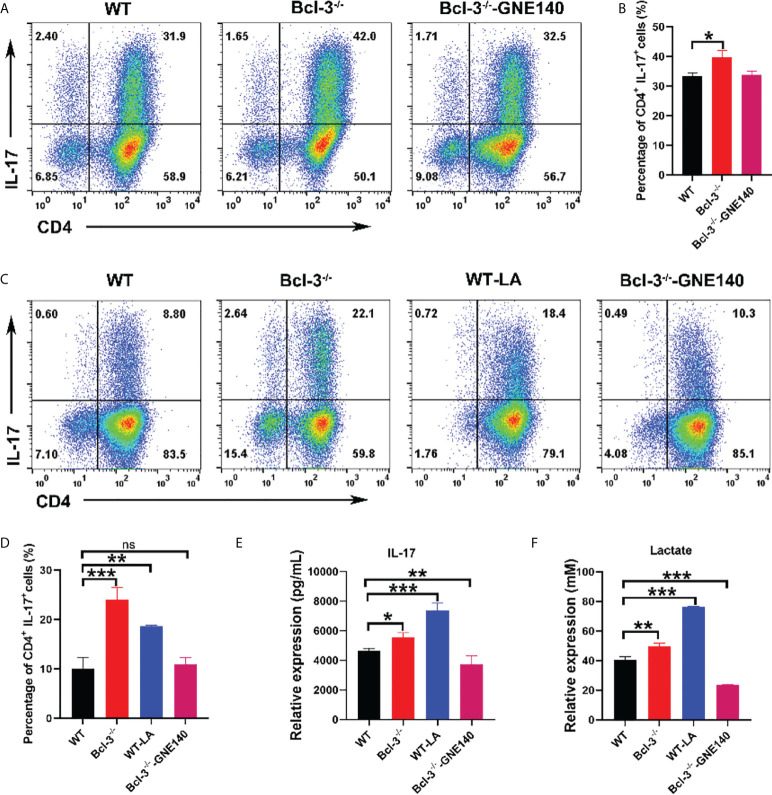
Bcl-3 regulates the differentiation of Th17 cells by lactate. **(A)** Th17 cells from the spleen lymphocytes of WT mice and Bcl-3^-/-^ mice induced using *in vitro* polarization assay, 10 μM GNE-140 was added to Bcl-3^-/-^ group for three days, and Th17 cells were analyzed by FACS. **(B)** Statistical analysis of Th17 cells. **(C)** Th17 cells from the spleen lymphocytes of WT mice and Bcl-3^-/-^ mice induced using *in vitro* polarization assay, 10 μM GNE-140 was added to Bcl-3^-/-^ group, 25 mM lactate were added to WT group for three days, and Th17 cells were analyzed by FACS. **(D)** Statistical analysis of Th17 cells. **(E)** IL-17 secretion in supernatant of Th 17 cells were detected by ELISA. **(F)** Lactate secretion in supernatant of Th 17 cells were detected by lactate detection kit. n = 3 for each group. Data above are representative of 3 independent experiments. *P <0.05, **P <0.01, ***P <0.001, “ns”, no significance.

The lactate dehydrogenase inhibitor GNE-140 was added to Bcl-3^-/-^ group to identify that it modulates the differentiation of Th17 cells. The results revealed that GNE-140 could decrease the percentage of IL-17^+^ CD4 T cells (**Figures 3A, B**), which suggests the suppressor role of GNE-140 on Th17 differentiation in Bcl-3^-/-^ mice. To further demonstrate the role of lactate in Th17 cell differentiation, LA was added to induced Th17 cells derived from WT mice, and the lactate dehydrogenase inhibitor GNE-140 was added to Bcl-3^-/-^ group. The results revealed that Th17 cells of WT mice supplemented with LA secreted significantly more IL-17 (**Figures 3C, D**). We also used an ELISA kit to detect IL-17 secretion in the supernatant. The findings were in line with the flow cytometry results. IL-17 secretion by Th17 cells of Bcl-3^-/-^ mice and by Th17 cells of WT mice supplemented with LA was significantly higher than normal WT mice (**Figure 3E**). LA contents were measured in cell supernatant by LA kit. It was found that higher LA contents were secreted by Th17 cells in the Bcl-3-/- group compared to WT group. Furthermore, adding GNE-140 to Th17 cells derived from Bcl-3^-/-^ mice could reduce LA (**Figure 3F**), suggesting the importance of LA in Th17 cell differentiation.

### Lactate regulates the energy metabolism of Th17 in Bcl-3-/- mice

**Figure 4:**
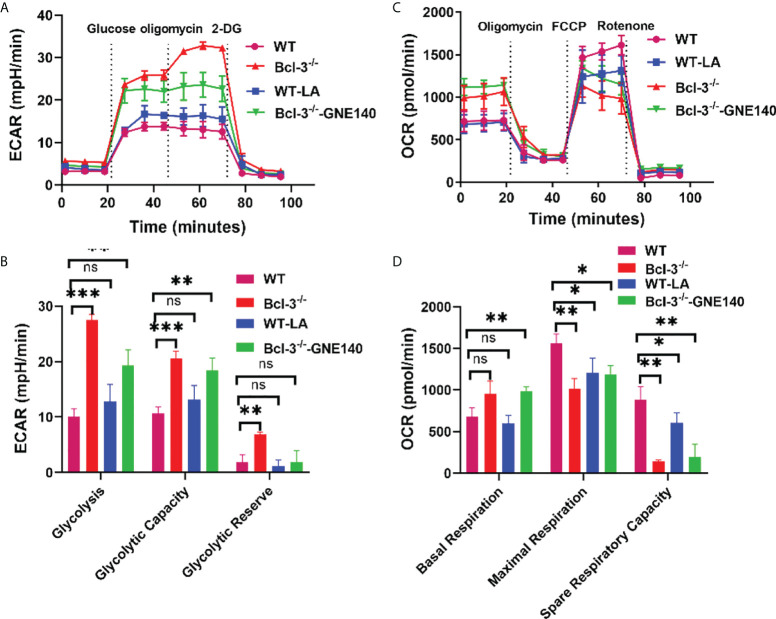
Lactate regulates the energy metabolism of Th17 in Bcl-3^-/-^ mice. Naïve CD4+ T cells were isolated from the spleen of Bcl-3^-/-^ and WT mice and induced Th17 differentiation by *in vitro* polarization assay, 10 μM GNE-140 was added to Bcl-3^-/-^ group, 25 mM lactate were added to the WT group for three days, and cells were detected the metabolic signals by seahorse XF24 extracellular flux analyzer. **(A)** Glycolytic pathway was detected by extracellular acidification rate (ECAR). **(B)** Statistical analysis of ECAR **(C)** mitochondrial respiration was detected by oxygen consumption rate (OCR). **(D)** Statistical analysis of OCR. n = 3 for each group. Data above are representative of 3 independent experiments. *P <0.05, **P <0.01, ***P <0.001, “ns”, no significance.

To further investigate whether LA regulates Th17, we added LA to *in vitro*- induced Th17 cells, and GNE-140 was added to Bcl-3^-/-^ group. A Seahorse XF24 extracellular flux analyzer measured the metabolic signal of Th17 cells. The results demonstrated that adding LA to Th17 cells from WT mice could increase ECAR but reduce the maximal respiratory rate induced by FCCP and reserve respiratory capacity (**Figure 4**). GNE-140, on the other hand, was able to inhibit ECAR of Th17 cells in Bcl-3^-/-^ mice which in turn improved the ultimate respiratory rate and respiratory reserve capacity induced by FCCP. These findings suggest that Bcl-3 may participate in mitochondrial energy metabolism and influence Th17 cell energy metabolism by regulating LA.

### Bcl-3 modulates gene-expression programs of Th17 cells

**Figure 5:**
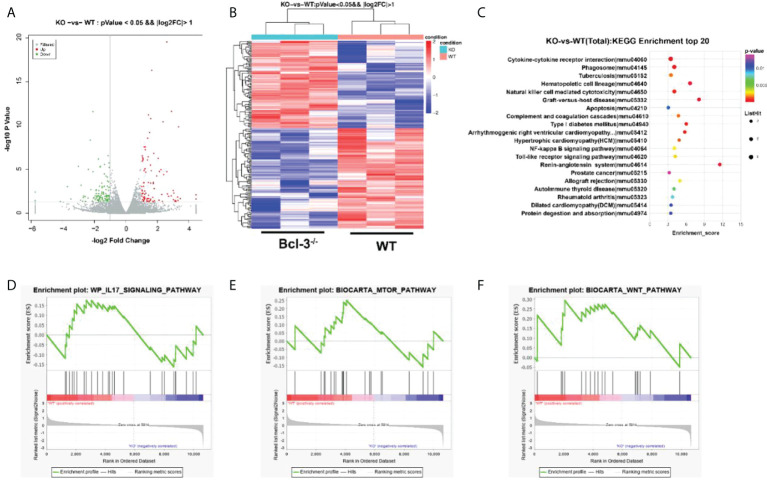
Bcl-3 modulates gene-expression programs of Th17 cells. **(A)** Volcano plot of transcriptomics data in Th17 cells from Bcl-3^-/-^ mice versus Th17 cells from WT mice. **(B)** Heatmaps representing genes differentially expressed (≥2-fold change, P < 0.05). **(C)** KEGG pathway analysis based on differentially expressed genes in Th17 cells of Bcl-3^-/-^ mice and Th17 cells from WT mice. The top 20 positively enriched pathways are shown in the bubble chart. **(D–F)** GSEA plots comparing Th17 cells from Bcl-3^-/-^ mice and Th17 cells from WT mice using ‘hallmark’ gene sets, showing the enrichment of IL-17 signaling, mTORC1 targets and Wnt pathways.

Transcriptome analysis of Th17 cells from WT and Bcl-3^-/-^ mice was performed to elaborate on the underlying cellular and functional processes. A heatmap of differentially expressed genes indicates that this differential gene expression in WT and Bcl-3^-/-^ mice corresponded to 198 probes. A total of 92 was up-regulated in Th17 from Bcl-3^-/-^ mice, while 106 were down-regulated in Bcl-3^-/-^ Th17 cells (**Figures 5A, B**). These genes are involved in immune response, metabolism, signal transduction, autophagy, and tube development. The most significantly enriched KEGG pathways included cytokine-cytokine receptor interaction, phagosome, hematopoietic cell lineage, and natural killer cell-mediated cytotoxicity (**Figure 5C**). The gene-set-enrichment analysis (GSEA) compared Th 17 cell gene expression in Bcl-3^-/-^ versus WT mice. It was found that WT subset was enriched for mTOR signaling, IL-17 pathway, and Wnt targets (**Figures 5D–F**).

### Bcl-3 deficiency alleviates the progress of EAE through lactate

**Figure 6:**
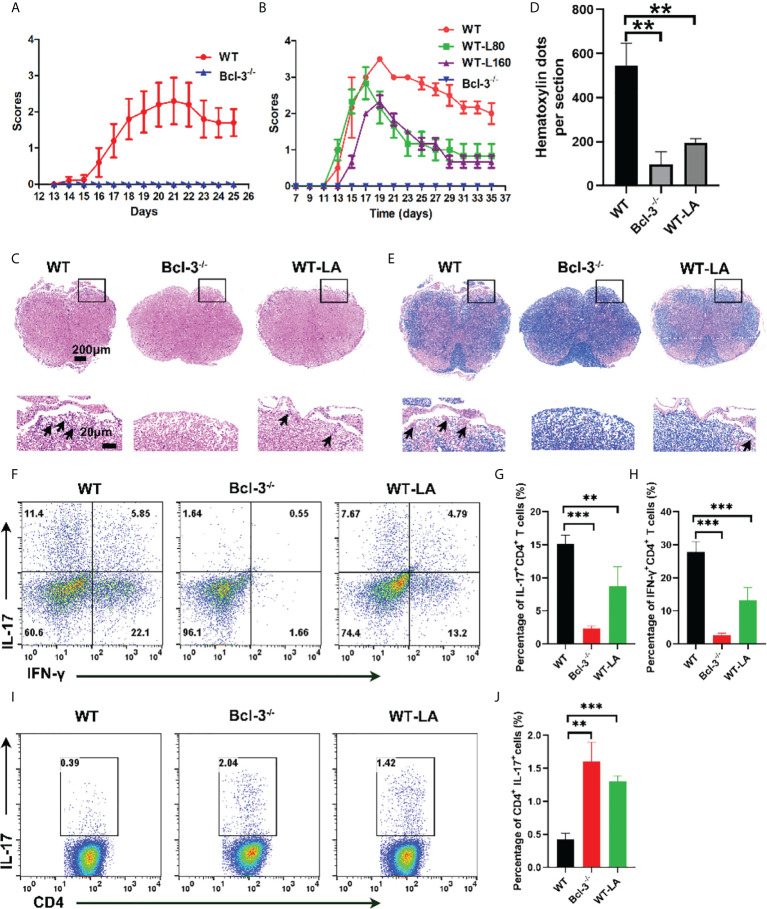
Bcl-3 deficiency alleviates EAE progression through lactate. **(A)** Clinical score of Bcl-3^-/-^ and WT mice after inducing EAE disease (n = 5). **(B)** Bcl-3^-/-^ and WT mice were induced EAE, and one group of WT mice received 160 mg/kg lactate i.p. three days later; clinical score of EAE is evaluated every other day. **(C)** Spinal cords were isolated from mice on day 18 after immunization and fixed for haematoxylin & eosin staining, and infiltration of lymphocytes was highlighted with arrow. **(D)** Quantitative analysis of disease severity. **(E)** Spinal cords were isolated from mice on day 18 after immunization and fixed for LFB staining to analyze demyelination highlighted by arrow. **(F)** Representative staining of Th17 cells in CNS on day 18 post immunization. Represented dots were gated on TCRβ^+^ CD4^+^ **(G–H)** statistical analysis of IL-17^+^ CD4^+^ cells and IFN-γ^+^ CD4^+^ cells in the CNS. **(I)** Representative staining of Th17 cells in draining lymph nodes on day 14 post immunization. Represented dots were gated on CD4^+^ **(J)** statistical analysis of IL-17^+^ CD4^+^ cells in the draining lymph nodes. Data above are representative of 3 independent experiments. **P <0.01, ***P <0.001.

To confirm the Bcl-3’s role in regulating Th17 through LA, we induced EAE disease in WT and Bcl-3 mice. In contrast, WT mice were supplemented with LA. The results demonstrate that LA supplementation reduced the incidence of EAE and demyelination (**Figures 6A–E**). In addition, we determined the infiltrating lymphocytes in spinal cord and brain of mice. It was found that the proportion of CD4^+^ IL17^+^ cells and the percentage of CD4^+^ IFN-γ^+^ cells in the spinal cord of Bcl-3^-/-^ mice and WT mice supplemented with LA was significantly reduced compared with WT mice (**Figures 6F–H**). However, the percentage of CD4^+^ IL17^+^ cells in the draining lymph nodes of Bcl-3^-/-^ mice and WT mice supplemented with LA was significantly higher than WT mice (**Figures 6I, J**). These findings show that Bcl-3 may modulate the function of Th17 *via* LA and thus affect EAE disease. Moreover, we examined whether Bcl-3 deficient mice have altered MOG _35-55_ specific CD4^+^ T cells by staining in non-polarized lymph node cells through MHC-Class II (I-Ab) MOG _35-55_ specific tetramer. The results suggested that the percentage of MOG _35-55_ specific CD4^+^ T cells was higher in immunized Bcl-3^-/-^ mice than WT mice (**Supplementary Figure S4**). In addition, we also detected the Th17 cells priming in draining lymph nodes and spleen at different time of immunized mice. As shown in **Supplementary Figure S5**, the percentage of CD4^+^ IL17^+^ cells in the draining lymph nodes have no significant difference at 5 days post immunization, while the percentage of CD4^+^ IL17^+^ cells of Bcl-3^-/-^ mice were significantly higher than that in WT mice as time progressed. The results were similar for the spleen. These data imply that Bcl-3 depletion may hampered migration of Th17 cells across the blood-brain barrier into the CNS.

### Bcl-3 regulates the mTOR signaling pathway by interacting with Raptor

We identified several metabolism-related signal proteins in Th17 cells to investigate the regulatory mechanism of Bcl-3 on energy metabolism and Th17 function *via* LA. As shown in **Supplementary Figures S6A, B**, the results indicated that compared to WT mice, Bcl-3 depletion reduced the expression of p-p70 and p-Akt in Th17 cells while not affecting the expression of mTOR, Raptor, and Hif1α. The expression of these proteins decreased after the lactate dehydrogenase inhibitor GNE-140 addition to Bcl-3^-/-^ Th17 cells, but there was no discrete difference in the expression of p-p70 and p-Akt. Simultaneously, we measured the expression of monocarboxylate transporter 1 (MCT1) and lactate dehydrogenase A (LDHA). Similarly, it was established that Bcl-3 depletion increased the expression of MCT1 and LDHA in Th17 cells as compared to WT mice. The expression of these proteins was increased after lactate was added to Th17 cells from WT mice. However, adding GNE-140 can reduce their expression in Bcl-3 depleted Th17 cells. Similar results were obtained by RT-PCR analysis (**Supplementary Figures S6C, D**). Therefore, the data suggest that Bcl-3 might influence Th17 cell differentiation by modulating the mTOR signaling pathway and LA expression.

**Figure 7:**
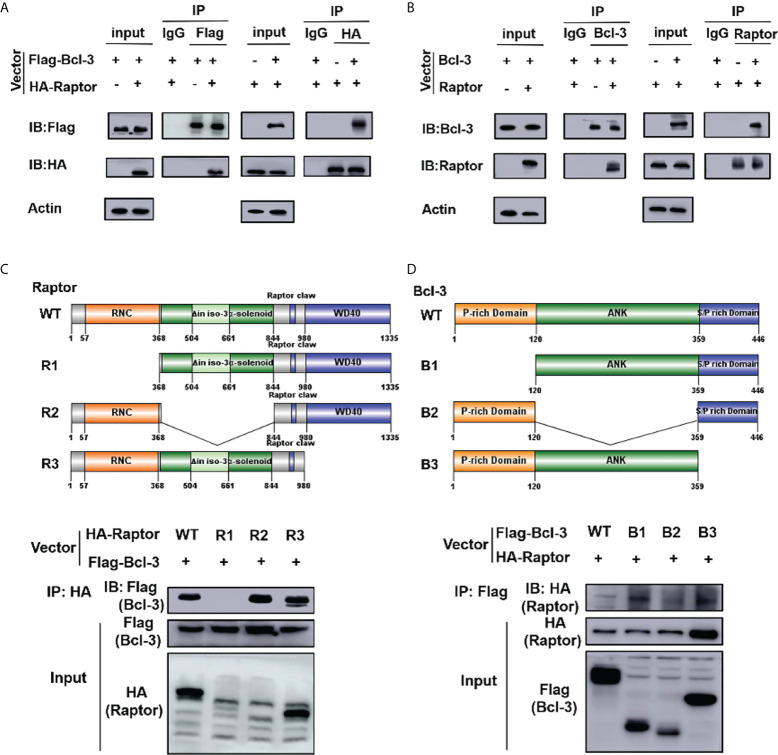
Bcl-3 interact with Raptor. **(A)** Flag-Bcl-3 interacted with HA-Raptor in 293T cells. The cells were co-transfected with Flag-Bcl-3 and HA-Raptor plasmids for co-IP assays using the anti-Flag and anti-HA antibodies, respectively. **(B)** Endogenous Bcl-3 interacted with endogenous Raptor in 293T cells detected by co-IP assays. **(C)** RNC domain of Raptor is needed for Raptor to interact with Bcl-3. 293T cells were transduced with plasmids expressing WT or different deletion mutants of HA-Raptor together with Flag-Bcl-3 vector for co-IP assays. **(D)** The ID domain of ANK is required for Bcl-3 to interact with Raptor. 293T cells were transduced with vectors expressing WT or different deletion mutants of Flag-Bcl-3 vectors together with the HA-Raptor vector for co-IP assays. Data above are representative of 2 independent experiments.

IP experiment was performed to clarify the influence of Bcl-3 on mTOR by determining the interaction among Bcl-3 and Raptor, a key protein of mTOR. We co-transfected Flag-Bcl-3 and Ha-Raptor into 293T cells and used co-IP to detect their potential interaction, followed by western-blot assays. Bcl-3 could interact with Raptor, as shown in **Figure 7A**. The interaction between endogenous Bcl-3 and Raptor was observed in 293T cells through a similar experiment (**Figure 7B**). We produced WT and deletion mutant Raptor with different flag markers. We co-transfected them with Flag-Bcl-3 vectors into 293T cells for co-IP assays to investigate the interaction domain between Raptor and Bcl-3. The findings suggested that Raptor’s RNC domain is necessary for their interaction with Bcl-3 (**Figure 7C**). We again used the co-IP assay to find Bcl-3 domain that interacts with Raptor by co-transfecting HA-Raptor vectors and vectors expressing different Flag-tagged deletion mutants of Bcl-3 into 293T cells with co-IP assays. The finding explored the interaction of Bcl-3’s ANK domain with Raptor (**Figure 7D**)

## Discussion

In this study, we explain the influence of Bcl-3 on Th17 cell function regulation through lactate production and the improved effect of lactate supplementation on the EAE model in mice. Bcl-3 might interact with Raptor to regulate mTORC1 mediating glycolytic metabolism and assure its pathogenicity under EAE conditions.

Th17 cells have protective effects under normal circumstances but can be pathogenic in autoimmune diseases (23, 24). We revealed that Bcl-3 depletion reduces the percentage of CD4^+^ T cells while promoting Th17 cells differentiation, IL-17 production, and RORγt expression. Moreover, we identified that Bcl-3 depletion protects mice from EAE. The results may corelated to the decreased expression of acetyl CoA carboxylase (ACC) and fatty acid synthase (FASN) in Bcl-3 deficient mice because the high activity of acetyl-CoA carboxylase 1 (ACC1) and FASN were associated with the pathogenicity of Th17 cells (19). ACC1 inhibition might block the differentiation of CD4^+^ T cells to Th17 cells, resulting in the amelioration of EAE progression (25). We also found that Bcl-3 could interact with Raptor. Deletion of raptor in Th17 cells leads to the defect of T-bet expression, resulting in reduced number of IL-17-IFN-γ^+^ cells and reduced the inflammation and clinical manifestations of central nervous system in EAE model (26). In addition, the resistance of Bcl-3^-/-^ mice against EAE may due to the decreased IFN-γ^+^CD4^+^ T cells and Th17 cells in the spinal cord. According to these findings, Bcl-3-depleted T cells could not cause T cell transfer-induced colitis or EAE (9). This protection against disease may be contributed by an increase in Th17 cells number and a decrease in Th1 cells that produce cytokines IFN-γ and GM-CSF (9). However, the specific overexpression of Bcl-3 in T cells will impair the development of Th2, Th1, and Th17 cells, leading to resistance to EAE (10). Another study suggests that low Bcl-3 expression is associated with an increased risk of multiple sclerosis (27), indicating the complexity of Bcl-3 action.

Metabolic rewiring is important in T cells activation and differentiation and in aerobic glycolysis, which converts glucose to lactate. It is considered an important marker of activated T cells (28–30), but its specific functions are still unknown (29). Glucose is the primary source of carbon, and it has a significant impact on T cell development, proliferation, and function (28, 31, 32). However, the exact effect of glycolysis on T cell responses is unknown. Activated T cells, including Th17 cells, use glucose metabolism to meet the metabolic demands of rapid proliferation and biosynthesis prerequisite for cellular growth and differentiation (33). However, in the absence of Bcl-3, Th17 cells showed decreased mitochondrial energy respiration and increased anaerobic metabolism. The results could be explained by the downregulation of the mTOR signaling pathway in Th17 cells. As mTOR is a key protein regulating the metabolism of sugar, lipid and protein. It is primarily found in the form of mTORC1 and mTORC2 complexes (34). mTOR and its key complex molecules play an important role in developing and differentiating T cells in the thymus (35). Raptor is a mTORC1 complex protein whose absence in mice impaired the Th1, Th2, Th17, and Treg differentiation (35). The dynamic regulation of metabolism and mTORC1 activation was involved in the development of thymocytes. Raptor deficiency disrupts the balance of oxidative and glycolytic metabolism, which impairs the progression of αβ T cells and promotes the development of γδT cells (36). Silencing mTOR or Raptor with shRNA promotes Tfh cell differentiation in activated T cells (37).

Although lactate was considered a waste product of glucose metabolism, now evidence shows its important role in modulating various biological processes, including macrophage polarization, Th cell differentiation, and tumor immune surveillance (38). Cytotoxic and other effector T cells rely heavily on glycolysis for proliferation and cytokines production and thus become inactive when glucose levels are low and lactate concentrations are high (39). The significantly higher levels of lactate in Th17 cells of Bcl-3 depleted mice might be due to upregulation of glycolytic metabolism and MCT1 expression, as MCT1 plays an important role in the uptake and transportation of lactate into cells, which is required for its function (40). Our subsequent research indicates that lactate could promote the differentiation of the Th17 cells and improve their ability of anaerobic metabolism, possibly due to the increased LDHA expression because LDHA is a rate-limiting enzyme with a high affinity for pyruvate and converts it to lactate. However, in the absence of LDHA, glucose metabolism in activated CD4^+^ T cells shifts from aerobic glycolysis to oxidative phosphorylation (41).

Similarly, L-lactate is important in regulating the tolerance of commensal bacteria that produce LA and are known to reduce inflammation in the gastrointestinal tract, such as lactobacilli and bifidobacteria (42). Therapy based on L-lactate is gaining attention and is being considered for a variety of other treatments, including pancreatitis (43), hepatitis (43), dengue fever (44). In this study, we demonstrated that lactate could alleviate the development of EAE in WT mice. In addition, the proportion of CD4^+^ IL17^+^ cells and CD4^+^ IFN-γ^+^ cells in the spinal cord of Bcl-3^-/-^ mice and LA supplemented WT mice was significantly lower than WT group. However, whether lactate regulates EAE is Th17 specific still needs further verification by adoptive transfer EAE experiments for the limitation of MOG_35-55_ induced EAE. Lactate may influence the function of Th17 cells through other cells, such as Th1 cells and Treg cells. It was reported that loss of Bcl-3 allowed for the conversion of pathogenic GM-CSF-expressing Th1 cells into non-pathogenic Th17-like cells in the context of EAE (9). Conditional overexpression of Bcl-3 can cause Treg dysfunction in mouse T cells, which is also important for the regulation of Th17 cells (10). We also found that the percentage of Th17 cells primed well in the secondary lymphoid organs of Bcl3^-/-^ mice upon MOG_35-55_ immunization while the presence of these cells in the CNS was lacked, which may be explained by following mechanisms: Bcl-3 depletion may hamper the migration of Th17 cells across the blood-brain barrier into the CNS. Deficiency of Bcl-3 may influence the survival of these Th17 cells if at all they enter the CNS and don’t make it long enough to induce pathological neuroinflammation. The reactive Th17 cells that entered into the CNS exhibit less pathogenic phenotype due to Bcl-3 loss, etc. Whether lactate alleviates the development of EAE by the same mechanism as Bcl-3 deserves to be further explored in our following study.

## Conclusion

In the present study, we first explain the metabolic regulatory role of the oncoprotein Bcl-3. We show that Bcl-3 regulates Th17 cell function by influencing lactate production and that lactate supplementation in mice alleviates the EAE model. Bcl-3 may interact with Raptor to regulate mTORC1 mediating glycolysis metabolism and assuring the pathogenicity of Th17 cells in the EAE context. Our findings reveal a potential regulatory mechanism that shapes adaptive immunity.

## Data availability statement

The data presented in the study are deposited in the NCBI repository, accession number PRJNA855272.

## Ethics statement

The animal study was reviewed and approved by Xinxiang Medical University.

## Author contributions

HW and HL conceived the study and wrote the manuscript. HL and LZ performed most of the experiments and analyzed the data YY designed the domain of Raptor and Bcl-3. ZH and CG performed some of the *in vivo* experiment. LH and XN performed the histopathology study on the spinal cord samples. CZ provided expertise and advice. HW oversaw the project. All authors contributed to the article and approved the submitted version.

## Funding

This work was funded by the National Natural Science Foundation of China (Grant No.81901590, 81871309).

## Conflict of interest

The authors declare that the research was conducted in the absence of any commercial or financial relationships that could be construed as a potential conflict of interest.

## Publisher’s note

All claims expressed in this article are solely those of the authors and do not necessarily represent those of their affiliated organizations, or those of the publisher, the editors and the reviewers. Any product that may be evaluated in this article, or claim that may be made by its manufacturer, is not guaranteed or endorsed by the publisher.
