# Supplementary material for: Bcl-3 regulates the function of Th17 cells through raptor mediated glycolysis metabolism
Source: Front Immunol. 2022 Sep 9;13:929785. doi: 10.3389/fimmu.2022.929785 (PMC9500237; doi:10.3389/fimmu.2022.929785)
Supplement: Supplementary file 1 [file DataSheet_1.docx]

**Bcl-3 regulates the function of Th17 cells through Raptor mediated glycolysis metabolism**

*Hui Liu^1†^, Lin Zeng^1†^, Yang Yang^2†^, Zhen Huang^1^, Chunlei Guo^1^, Liwenhui Huang^1^, Xinqing Niu^1^, Chenguang Zhang^1^, Hui Wang^1*^*

^1^ Henan Key Laboratory of Immunology and Targeted Drug, Henan Collaborative Innovation Center of Molecular Diagnosis and Laboratory Medicine, School of Laboratory Medicine, Xinxiang Medical University, Xinxiang 453003, Henan, China

^2^ Department of Translational Medicine Center, The First Affiliated Hospital of Zhengzhou University, Zhengzhou, China.

*** Correspondence:**Corresponding Author
wanghui@xxmu.edu.cn

*^†^* These authors contributed equally to this work

**Keywords:** Bcl-3, Th17, Lactate, Raptor, Glycolysis metabolism

**Figure S1**


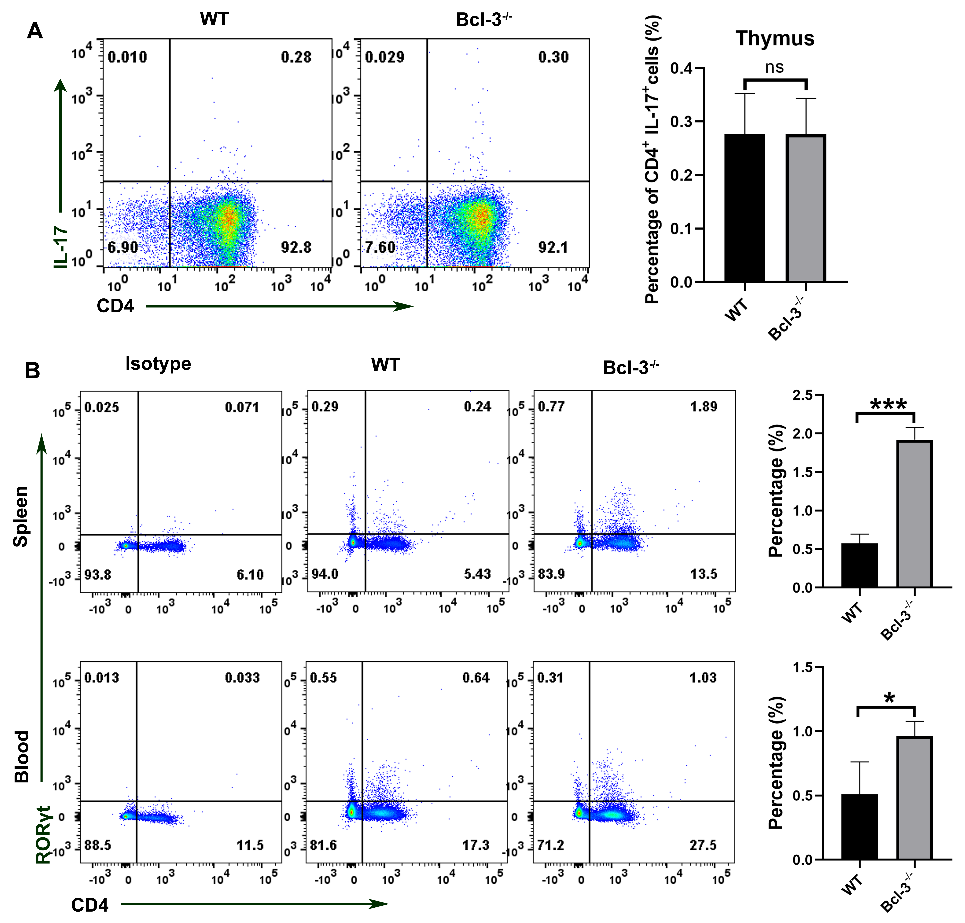


**Figure S1**. Bcl-3 depletion significantly increase RORγt expression in blood and spleen. (A) Lymphocytes were isolated from Thymus of Bcl-3^-/-^ and WT mice and analyzed by FACS. (B) Lymphocytes were isolated from spleen and blood of Bcl-3^-/-^ and WT mice and RORγt expression was analyzed by FACS.

**Figure S2**


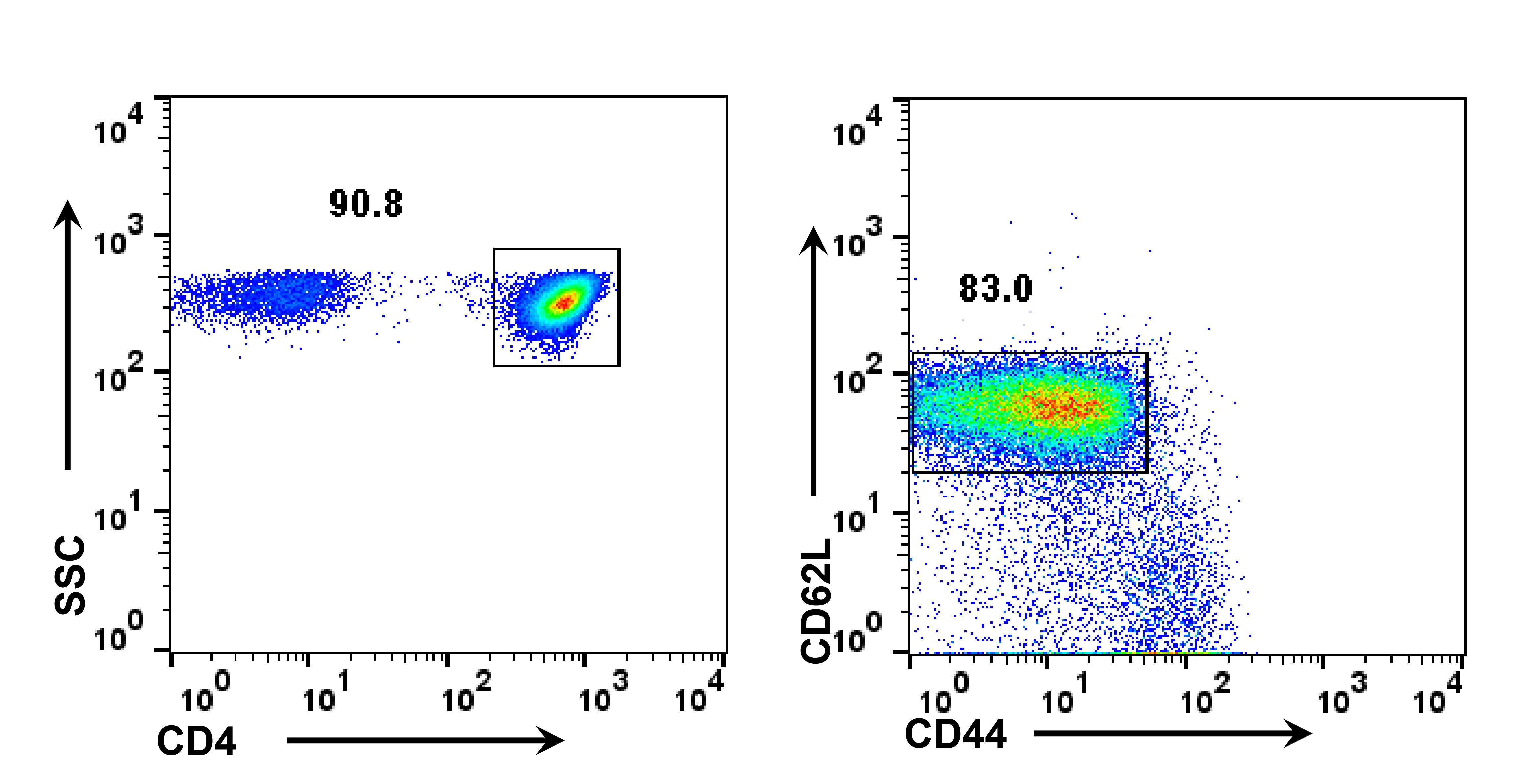


**Figure S2.** The post-sort purity of naive CD4^+^ T cells detected by FACS.

**Figure S3**

**
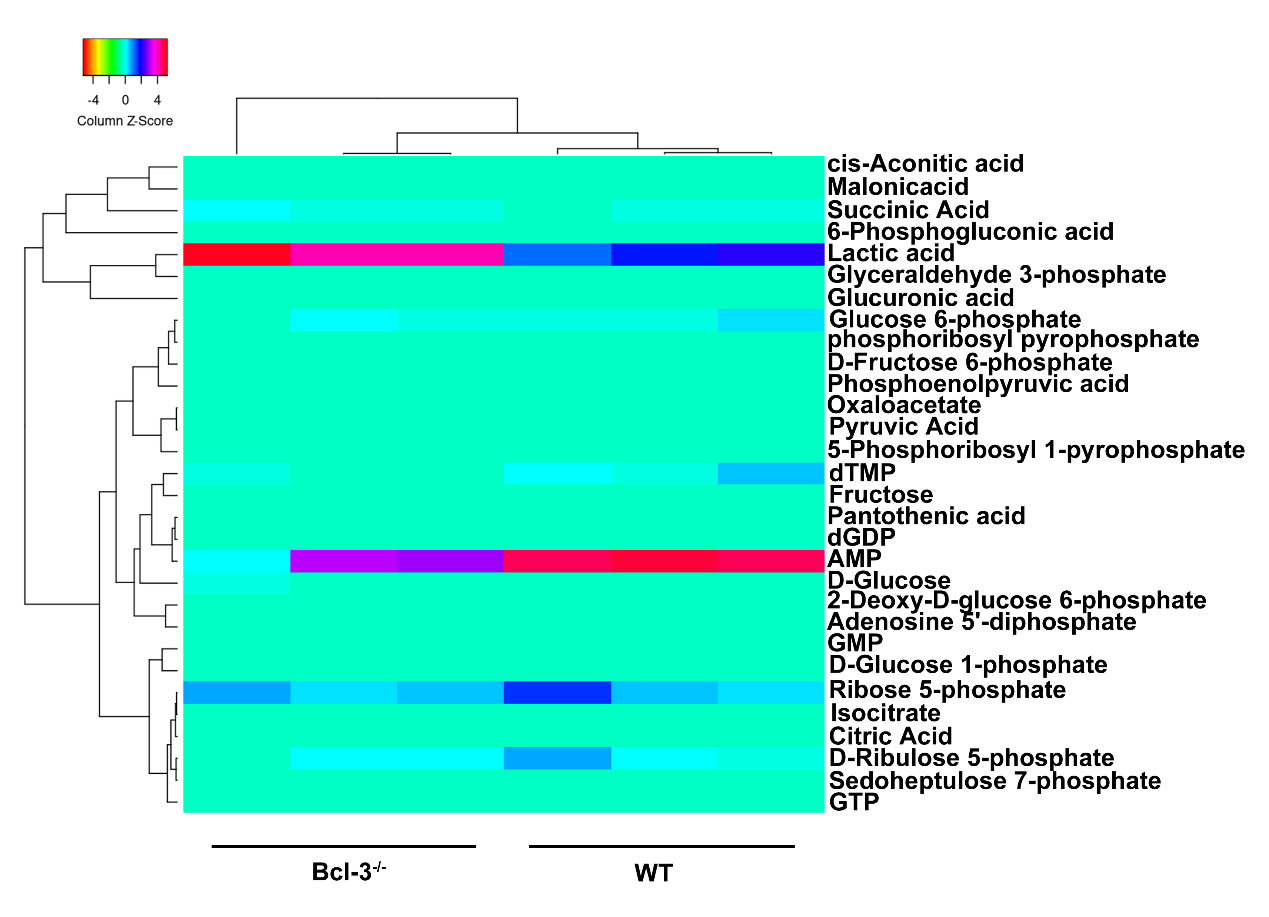
**

**Figure S3**. Bcl-3 depletion increased the expression of lactate of Th17 cells. Naïve CD4^+^ T cells from the spleen lymphocytes of WT mice and Bcl-3^-/-^ mice and induced differentiation to Th17 cells for targeted glycometabolomic detection and analysis; the heatmap of metabolites was indicated.

**Figure S4**


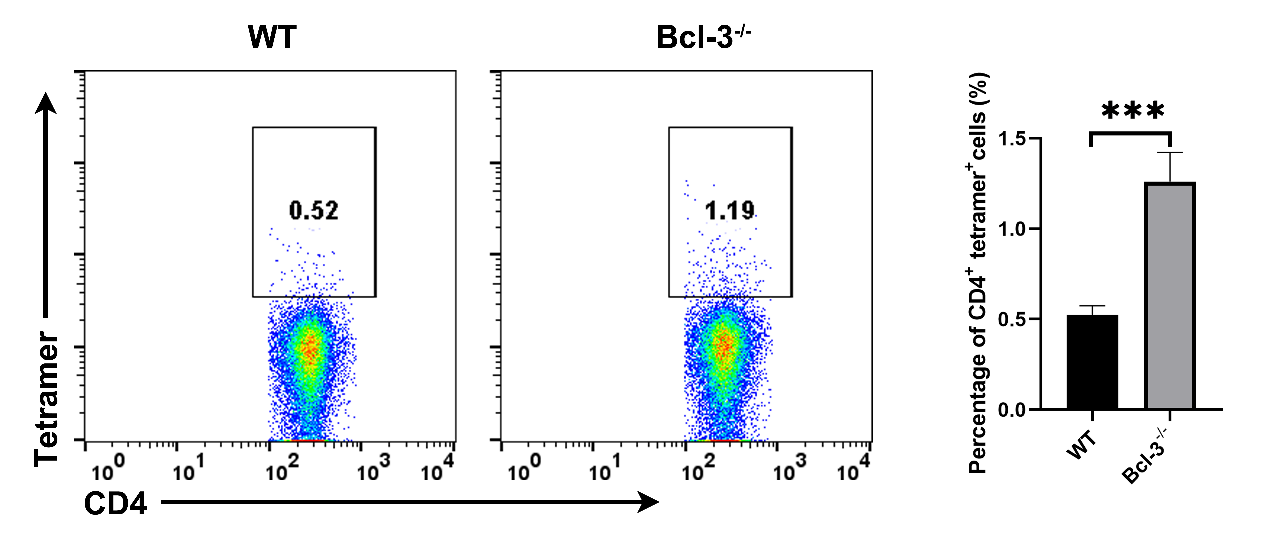


**Figure S4.** Bcl-3 depletion increased the percentage of MOG 35-55 specific CD4^+^ T cells in draining lymph nodes in immunized mice. Bcl-3^-/-^ and WT mice were induced EAE, then draining lymph nodes were isolated from mice on day 14 after immunization and stained by MHC-Class II (I-Ab) MOG_35-55_ specific tetramer (n=5). ***P ＜0.001.

**Figure S5**


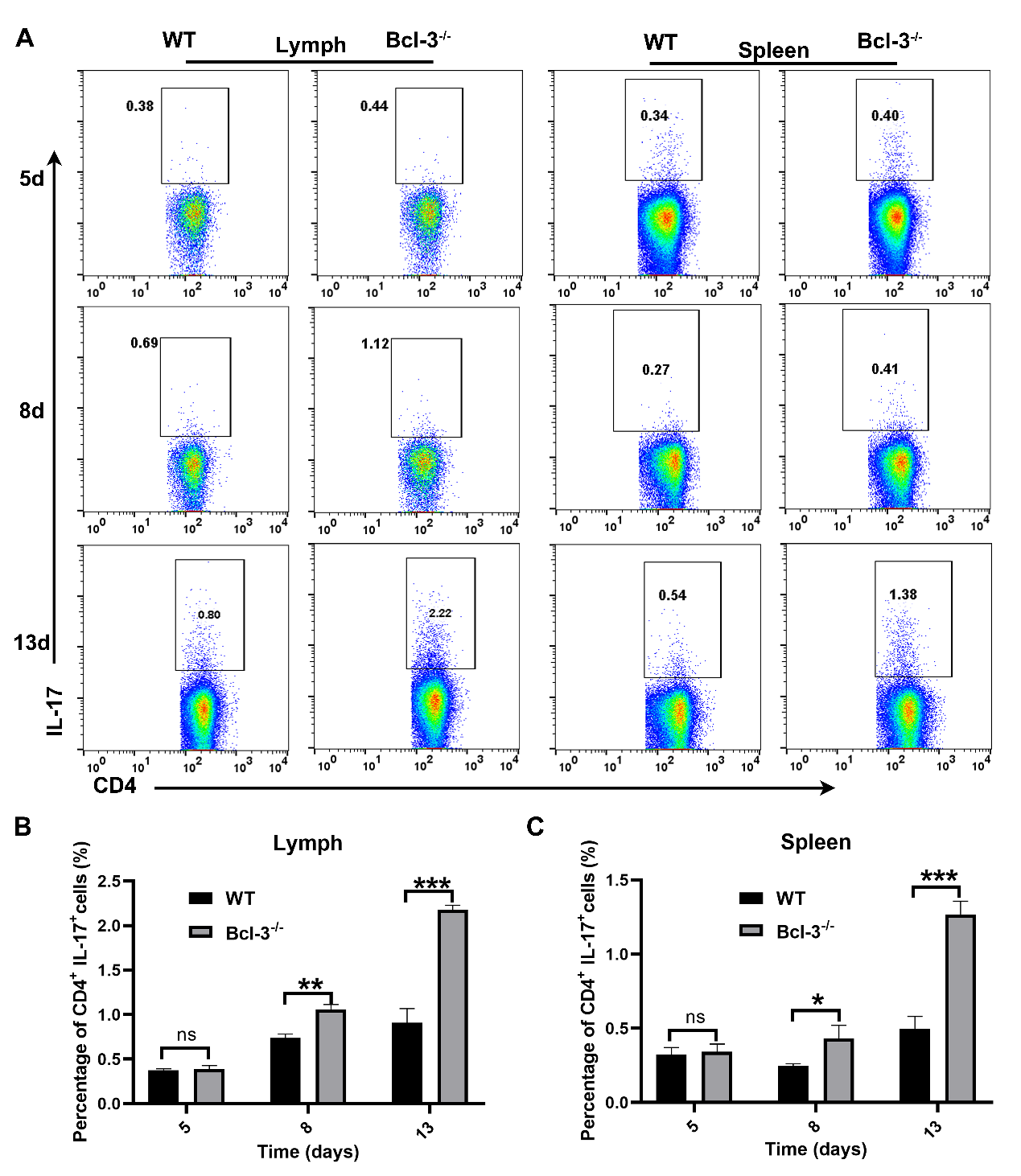


**Figure S5.** Bcl-3 depletion promotes Th17 cells priming in draining lymph nodes and spleen in immunized mice. Bcl-3^-/-^ and WT mice were induced EAE, then spleen and draining lymph nodes were isolated from mice on day 5, 8 and 13 after immunization. (A) Representative staining of Th17 cells in spleen and draining lymph at different time point. Represented dots were gated on CD4^+^ (n=5). (B-C) statistical analysis of IL-17^+^ CD4^+^ cells in the lymph nodes and spleen. *P ＜0.05, **P ＜0.01, ***P ＜0.001.

**Figure S6**


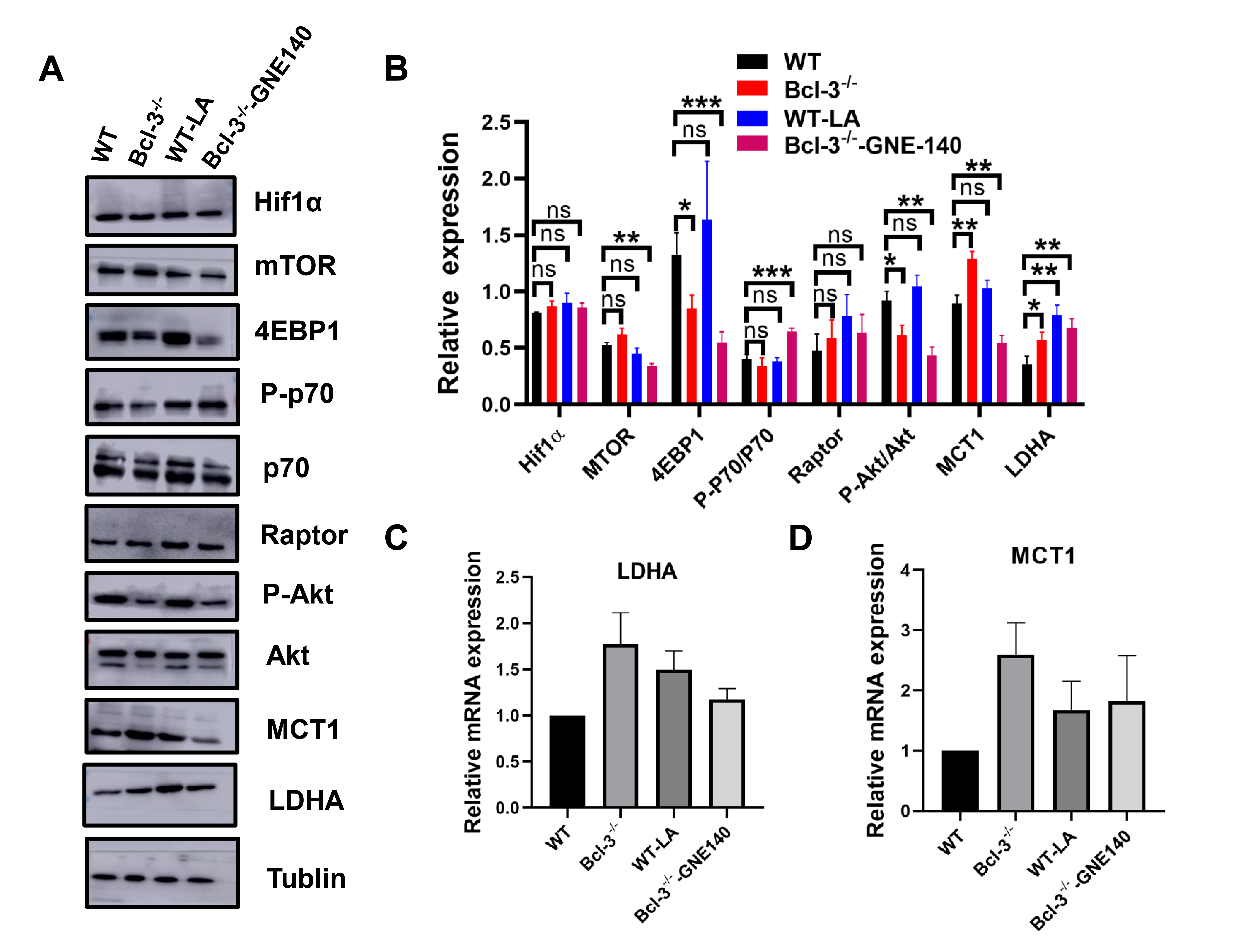


**Figure S6**. Bcl-3 regulates mTOR signaling pathway. Naïve CD4^+^ T cells were isolated from the spleen of Bcl-3^-/-^ and WT mice and induced Th17 differentiation by in vitro polarization assay, 10 μM GNE-140 was added to Bcl-3^-/-^ group and 25 mM lactate were added to the WT group for three days, then the cells were lysed for extracting proteins or RNA. (A-B) The proteins Hif1α, mTOR, MCT1, LDHA, Raptor, 4EBP1 and phosphorylated (p-) and total p70 and Akt signaling proteins in whole-cell lysates were determined by Western blot. (C-D) RT-PCR analysis for mRNA expression of LDHA and MCT1.
